# Russian version of BRIEF2 Teacher Forms: validation study in typically developing children aged 5 to 7 years old

**DOI:** 10.3389/fpsyg.2023.1260107

**Published:** 2023-11-21

**Authors:** Margarita Gavrilova, Margarita Aslanova, Kristina Tarasova, Yuri Zinchenko

**Affiliations:** Faculty of Psychology, Lomonosov Moscow State University, Moscow, Russia

**Keywords:** executive function, higher mental functions, BRIEF2, typically developing children, confirmatory factor analysis, Russian version of BRIEF2

## Abstract

This study aimed to examine the psychometric properties of the Russian version of the Behavioral Rating Inventory of Executive Function^®^, Second Edition (BRIEF2) Teacher Forms and investigate its dimensional structure. The sample consisted of 178 typically developing children aged 5 to 7 years. Internal reliability analysis indicated high reliability (from 0.87 to 0.95) for the three composite indices and the Global Executive Composite across all age groups. Confirmatory factor analysis revealed that the dimensional structure of the Russian version of BRIEF2 was different among children aged 5 and 6 years compared to children aged 7 years, which is probably because, in Russia, the transition from kindergarten to school takes place upon reaching the age of 7. The results confirm that the Russian version of the BRIEF2 Teacher Form can be used as a valid tool to assess EF in 5- and 6-year-old children, provided that the three BRIEF2 indices are used for clinical interpretation. For 7-year-old children, the BRIEF2 was found to be an insufficiently valid tool to assess executive function.

## Introduction

Although the definition of executive functions (EF) has long been debated, it may broadly be viewed as those “cognitive processes that are required for the conscious, top-down control of action, thought, and emotions and that are associated with neural systems involving the prefrontal cortex” (Müller and Liben, 2015, p. 271). Core EF skills include inhibitory control (resisting habits, temptations, or distractions), working memory (holding and using information), and cognitive flexibility (Miyake et al., 2000). These skills are extensively developed in the early years of a child's life, influencing a child's future outcomes (Tvardovskaya et al., 2022).

For the first time, the term “executive” appeared in the studies of the Soviet neuropsychologist Luria (1966), who was one of the founders of the Cultural-Historical Theory (Goldstein et al., 2014). As a neuropsychologist, he analyzed the functioning of various brain regions during clinical work with victims of brain injury in World War II. Luria stated that the frontal lobes are responsible for governing behavior and performing complex activities, based on the observation of many patients with injuries localized in this region of the brain (Luria, 1966). Thus, in 1966, he gave a definition of EF that is very close to the one shared by many researchers today: “…Syntheses underlying own actions, without which goal-directed, selective behavior is impossible” (Luria, 1966, p. 224). Higher mental functions, one of the central concepts of cultural-historical theory, are the equivalent of the EF concept (Ardila, 2008). The main difference in this understanding is that Vygotsky and Luria insisted that the executive processes occur through cultural means (e.g., symbols, speech, and writing) (Luria, 1966; Vygotsky, 2016).

EF skills play a key role in the development of academic skills, including word reading, vocabulary, and comprehension of both oral and written language, the acquisition of initial mathematical concepts, the formation of speech skills, and high school performance (Blair and Razza, 2007; Blankson et al., 2012; Gagne, 2017; Veraksa and Veraksa, 2021; Dolgikh et al., 2022; Veraksa et al., 2022). Identifying deficits in children's EF skills is important in preschool and school education (Trushkina and Skoblo, 2022). Assessments using professional tools provide the necessary support for children to develop their EF skills (Brofman et al., 2022). Behavioral rating screening may be a more affordable solution, especially in medium- to large-group settings (kindergarten or school), and the Behavioral Rating Inventory of Executive Function^®^, Second Edition (BRIEF2; Gioia et al., 2015) is one such tool.

### Behavioral rating inventory of executive function

BRIEF2 is a behavioral rating scale designed to assess the “real world” behaviors associated with EF at ages 5–18 years (parent and teacher forms) and ages 11–18 years (self-report form) (Gioia et al., 2015). The BRIEF2-Teacher Form consists of 63 items constituting nine subscales: Inhibit, Self-Monitor, Shift, Emotional Control, Initiate, Working Memory, Plan/Organize, Task-Monitor, and Organization of Materials. Three composite indices are derived from these subscales: the Behavior Regulation Index (BRI), the Emotion Regulation Index (ERI), and the Cognitive Regulation Index (CRI). The Global Executive Composite (GEC) is an overall EF score comprising three indexes.

BRIEF2 is widely used worldwide in clinical, psychoeducational, and research settings (Jiménez and Lucas-Molina, 2019; Robertson et al., 2020; Cumming et al., 2023) and has been successfully validated in several languages (Muñoz and Filippetti, 2021; Liu et al., 2022; Parhoon et al., 2022; Moura et al., 2023). Many of the existing studies have mainly been conducted on samples from North America (Jacobson et al., 2020) and Europe. However, several studies have validated BRIEF2 as also applicable to non-Western populations: China (Liu et al., 2022), Iran (Parhoon et al., 2022), Chile (Muñoz and Filippetti, 2021), the Dominican Republic (Jiménez and Lucas-Molina, 2019).

### Cultural and educational context in Russia

As with other diagnostic tools, the use and validation of BRIEF2 in Russia are marked by several country-specific peculiarities. Of particular importance are the state's multiculturalism and the particularities of the education system. Russia is a multicultural country requiring special conditions for adapting, validating, or standardizing diagnostic tools. There are 190 nationalities in the country, with 80% of the population being Russian (Malkov, 2020). To date, 24 of the Republics of Russia have established republican languages recognized as co-official with Russian (Jankiewicz et al., 2020). Multicultural and multilingual contexts require the translation of a tool into various national languages (Buryat, Dagestan, Tatar, Yakut, etc.).

From the perspective of the education system, an essential issue in validating a tool is the difference in the age period of the transition from ECEC to primary school. In Russia, children start school when they are 7 years old. Before they start primary school, children are enrolled in kindergarten from 3 to 6 years of age. The kindergarten educational programs include classes in reading, numeracy, and writing. However, these activities are different from school classes. Thus, some BRIEF2 items may have differing views on the conditions of education for children before and after the age of 7.

### Present study

This study belongs to a body of research investigating the cross-cultural applicability of BRIEF2 and potential cultural differences in everyday children's EF behavior in different cultural contexts. The study aims to analyze the psychometric properties (reliability and validity) of the Russian version of BRIEF2 Teacher Forms in a sample of typically developing children aged 5–7 years and investigate its dimensional structure. In addition, this study explores the interrater reliability between children's BRIEF2 results evaluated by two teachers. The study was designed considering the peculiarities of Russia's cultural and educational context. First, due to the multilingual nature of the state, the validity evidence on the applicability of the Russian-language version of BRIEF2 in this study may extend mainly to monolingual regions. Second, the study involved children aged 5–7 years old to assess the applicability of the Russian-language version of BRIEF2 over existing differences in the age period of the transition from ECEC to primary school.

## Methods

### Participants

The study included 178 typically developing children aged 5–7 years. The participants attended publicly funded kindergartens and schools. Children aged 5 and 6 years attended kindergartens, and children aged 7 years attended the first grade of school. Forms were filled out by 34 kindergarten and 8 primary school teachers (Table 1).

**Table 1:** Study participants.

**Age group**	***N***	**Age**	**Girls (%)**
5-year-old children	53	5.26 (± 0.32)	51.3
6-year-old children	69	5.48 (± 0.33)	61.5
7-year-old children	56	5.37 (± 0.43)	60.5

### Procedure

The study was conducted in May–June 2023. In kindergartens, the BRIEF2 was completed by two preschool teachers for each child. In the first grade, the BRIEF2 was completed by a full-time teacher. The teacher was asked to assess 6–10 children who were selected randomly based on their order of appearance on the class list (every third child). This measure of sampling was driven by classroom conditions in Russia, where class sizes are typically ~25–30 students. If teachers completed the form for the whole class, the quality of completion would probably be reduced. The lists given to the teachers were prepared in advance, considering medical and psychological screening information (only children with typical development were included). Another selection condition was that the teacher should have been familiar with the selected children for at least 6 months.

## Measures

### A Russian version of the BRIEF2

A Russian version of the BRIEF2 Teacher Form was derived from the English version of the BRIEF2 (Gioia et al., 2015). The BRIEF2 was translated from English into Russian by two professional bilingual experts based on standard translation guidelines and cultural adaptation processes (e.g., Sousa and Rojjanasrirat, 2011). Both translators have experience translating diagnostic tools in the fields of early childhood education and psychology. In the translation process, points were noted where there could be differences in the educational processes of organizations in Russia and the USA. Then, similar questions were addressed to an American specialist in preschool and primary school education. For example, to improve the translation's accuracy, clarification was obtained concerning the “criteria” for well-organized written work of a 5- to 7-year-old child (BRIEF2, para. 21). It is highly probable that the Russian criteria would differ. Next, a series of peer discussions were held with six experts in child development psychology and EF. The discussions included working with the original English manual and two versions of the Russian translation to select the optimal phrasing and refine it. All specialists were proficient in both Russian and English. Then, a native English speaker fluent in Russian translated the Russian-language version into English, and this back-translation was assessed for inconsistencies. After the Russian version of the BRIEF2 had passed the checks mentioned above, focus group interviews were conducted with 12 teachers who work with typically developing children at kindergarten or school. They were asked to share their understanding of each BRIEF2 item and comment on the statements' clarity and comprehensibility. Once the problems identified in the focus group interviews had been fully addressed, the final Russian version of the BRIEF2 questionnaire was used for full psychometric testing.

### Data analysis strategy

First, the reliability of the BRIEF2 Russian version was assessed using (a) internal scale consistency (Cronbach's alpha) and (b) interrater reliability between two teachers' reports for a single child, as based on the Cohen's kappa coefficient and a paired samples *t*-Test. Second, Pearson's correlation analysis was performed to analyze the correlation between the nine subscales, the three indexes, and the GEC. Third, construct validity has been analyzed based on the internal structure using confirmatory factor analysis. A CFA was conducted for the total sample and separately for three age groups to test the model fit of the BRIEF2 3-factor and 9-factor models.

## Results

### Descriptive

Given the variation in educational settings for children aged 5, 6, and 7 years, the primary tool's psychometric properties were evaluated separately for each age group. Table 2 presents descriptions of BRIEF2 nine subscales separately for these age groups. All the data were provided based on the row values for each subscale.

**Table 2:** Descriptive statistics for BRIEF2 subscales and Cronbach's alpha coefficients.

	**5 year old children (*****n** =* **53)**					**6 year old children (*****n** =* **69)**					**7 year old children (*****n** =* **56)**				
	**Min**	**Max**	**Mean**	**SD**	α	**Min**	**Max**	**Mean**	**SD**	α	**Min**	**Max**	**Mean**	**SD**	α
Inhibit	1.00	2.88	1.59	0.49	0.88	1.00	2.88	1.59	0.49	0.84	1.00	2.38	1.38	0.35	0.80
SM	1.00	3.00	1.67	0.60	0.84	1.00	3.00	1.67	0.60	0.81	1.00	2.60	1.38	0.42	0.78
Shift	1.00	3.00	1.62	0.45	0.85	1.00	3.00	1.62	0.45	0.75	1.00	2.25	1.45	0.33	0.75
EC	1.00	2.88	1.62	0.49	0.89	1.00	2.88	1.62	0.49	0.89	1.00	2.38	1.40	0.32	0.70
Initiate	1.00	2.80	1.46	0.40	0.74	1.00	2.80	1.46	0.40	0.57	1.00	2.75	1.45	0.43	0.41
WM	1.00	1.63	1.59	0.49	0.89	1.00	2.75	1.74	0.47	0.84	1.00	2.38	1.39	0.38	0.88
Plan	1.00	3.00	1.54	0.46	0.84	1.00	3.00	1.54	0.46	0.84	1.00	2.50	1.46	0.45	0.84
TM	1.00	3.00	1.62	0.51	0.81	1.00	3.00	1.62	0.51	0.81	1.00	2.60	1.38	0.43	0.82
OM	1.00	2.80	1.55	0.48	0.75	1.00	2.80	1.55	0.48	0.87	1.00	2.38	1.38	0.35	0.81
BRI	1.00	2.81	1.58	0.51	0.95	1.00	2.75	1.82	0.51	0.87	1.00	2.49	1.38	0.36	0.83
ERI	1.00	2.88	1.55	0.46	0.95	1.00	2.63	1.54	0.49	0.91	1.00	2.31	1.43	0.30	0.83
CRI	1.00	2.62	1.56	0.40	0.94	1.00	2.66	1.67	0.40	0.92	1.00	2.37	1.38	0.35	0.94
GEC	1.00	2.66	1.53	0.41	0.97	1.00	2.53	1.58	0.49	0.95	1.00	2.39	1.39	0.33	0.96

Source: BRIEF2 (Russian version). (a) BRIEF2, Behavior Rating Inventory of Executive Function, Second Edition; (b) mean raw scores range from 1–3; (c) SM, Self-Monitor; EC, Emotional Control; WM, Working Memory; Plan, Plan/Organize; TM, Task Monitor; OM, Organization of Materials; BRI, Behavior Regulation Index; ERI, Emotion Regulation Index; CRI, Cognitive Regulation Index; GEC, Global Executive Composite; (d) α – Cronbach's alpha coefficient.

### Reliability/precision of the subscales

#### The internal consistency

The internal consistency was studied using Cronbach's alpha coefficients as a first metric of reliability, separately, for each age group. Cronbach's alpha reliability coefficient ranges between 0 and 1. George and Mallery (2003) suggest that an alpha coefficient value result be interpreted as follows: > 0.9 is excellent; > 0.8 – is good; > 0.7 is acceptable; > 0.6 is questionable; > 0.5 is poor, and < 0.5 is unacceptable.

In the sample of 5-year-old children, the results indicate that the internal consistency of all subscales was good or acceptable (see Table 2). Cronbach's alpha coefficients for each subscale ranged from 0.74 (Initiate) to 0.89 (Emotional Control and Working Memory), and the Cronbach's alphas for each index ranged from 0.95 (Shift) to 0.97. In the sample of 6-year-old children, the internal consistency was good or acceptable for all subscales except the Initiate. Cronbach's alpha coefficients for subscales ranged from 0.57 (Initiate) to 0.89 (Emotional Control), and the Cronbach's alphas for each index ranged from 0.87 to 0.95. In the sample of 7-year-old children, the internal consistency was, in general, lower than that in the previous two age groups. Cronbach's alpha coefficients for subscales ranged from 0.41 (Initiate) to 0.88 (Working Memory). The Cronbach's alphas for each index ranged from 0.83 to 0.97.

#### The interrater reliability

Next, interrater reliability has been studied based on the Cohen's kappa coefficient as a second metric of reliability and additionally tested using the paired samples *t*-test. This analysis was conducted only for 5- and 6-year-old children because, at these ages in the country of study, children attend kindergarten and have regular contact with two preschool teachers. Cohen's kappa coefficient, ranging from 0 to 1, assesses the measure of consistency between the scores of these two teachers from 0 to 1. According to McHugh (2012) recommendations for the interpretation of Cohen's kappa, the value of kappa ≤0.20 indicating no agreement; 0.21–0.39 is a minimal level; 0.40–0.59 is a weak level; 0.60–0.79 is moderate level; 0.80–0.90 is a strong level, and ≥0.90 is an almost perfect level of agreement.

Table 3 presents interrater reliability analysis results for two age groups separately. The lowest level of agreement among teacher reports for children aged 5 years was found in the Plan/Organize subscale. A weak level of agreement was observed for Shift, Initiate, Working Memory, and Organization of Materials. Other subscales showed moderate (Inhibit, Emotional Control, and Task-Monitor) or strong (Self-Monitor) levels of agreement. The level of agreement between teachers' reports for children aged 6 years is generally lower than that for children aged 5 years. The lowest level of agreement was found specifically with the Initiate. Weak agreement was observed on six of the tool's nine subscales (Inhibit, Shift, Working, Memory, Plan/Organize, Task-Monitor, and Organization of Materials). Only two subscales (Self-Monitor and Emotional Control) had moderate levels of agreement.

**Table 3:** Interrater reliability for BRIEF2 subscale scores reported by preschool teachers.

	**5-year-old children (*****n** =* **53)**			**6-year-old children (*****n** =* **69)**		
	κ	***Z***	***P***	κ	***z***	***p***
Inhibit	0.76	5.53	< 0.001	0.50	5.41	< 0.001
SM	0.81	6.45	< 0.001	0.72	8.77	< 0.001
Shift	0.52	3.90	< 0.001	0.49	5.42	< 0.001
EC	0.65	4.60	< 0.001	0.67	4.93	< 0.001
Initiate	0.50	3.69	< 0.001	0.27	3.02	< 0.001
WM	0.51	3.75	< 0.001	0.45	5.04	< 0.001
Plan	0.37	2.96	< 0.001	0.45	5.12	< 0.001
TM	0.73	6.03	< 0.001	0.40	4.77	< 0.001
OM	0.54	3.97	< 0.001	0.42	4.57	< 0.001

(a) Mean raw scores range from 1 to 3; (c) SM, Self-Monitor; EC, Emotional Control; WM, Working Memory; Plan, Plan/Organize; TM, Task Monitor; OM, Organization of Materials; BRI, Behavior Regulation Index; ERI, Emotion Regulation Index; CRI, Cognitive Regulation Index; GEC, Global Executive Composite; (b) α – Cronbach's alpha coefficient.

The paired samples *t*-test did not find significant differences between the two teachers' reported scores for either of the BRIEF2 subscales (*p* > 0.05).

#### Intercorrelations

A Pearson's correlation analysis was conducted to analyze the correlation between the nine subscales, the three indexes, and the GEC. The intercorrelation matrix showed that all nine subscales and index scores were moderately to highly correlated. Correlation coefficients ranged from 0.64 to 0.97. All correlations were significant at a *p*-value of < 0.001 (see Table 4).

**Table 4:** Intercorrelations among the nine subscales, three indexes, and GEC of the Russian version of the BRIEF2 teacher forms.

**Subscale**	**1**	**2**	**3**	**4**	**5**	**6**	**7**	**8**	**9**	**10**	**11**	**12**	**13**
Inhibit	—												
SM	0.872	—											
Shift	0.844	0.793	—										
EC	0.856	0.841	0.780	—									
Initiate	0.643	0.684	0.725	0.629	—								
WM	0.731	0.660	0.794	0.717	0.708	—							
Plan	0.838	0.789	0.818	0.829	0.710	0.779	—						
TM	0.822	0.780	0.807	0.812	0.716	0.779	0.770	—					
OM	0.832	0.725	0.829	0.737	0.636	0.774	0.773	0.839	—				
BRI	0.962	0.973	0.848	0.876	0.686	0.718	0.843	0.826	0.801	—			
ERI	0.901	0.885	0.940	0.946	0.725	0.800	0.880	0.854	0.829	0.923	—		
CRI	0.873	0.810	0.889	0.832	0.823	0.917	0.903	0.922	0.906	0.869	0.910	—	
GEC	0.944	0.933	0.929	0.914	0.798	0.838	0.924	0.891	0.872	0.968	0.976	0.956	—

(a) SM, Self-Monitor; EC, Emotional Control; WM, Working Memory; Plan, Plan/Organize; TM, Task Monitor; OM, Organization of Materials; BRI, Behavior Regulation Index; ERI, Emotion Regulation Index; CRI, Cognitive Regulation Index; GEC, Global Executive Composite; (b) all correlations are significant at level < 0.001.

### Construct validity

The underlying factor structure of the Russian version of the BRIEF2 Teacher Forms was explored using a confirmatory factor analysis (CFA) with maximum likelihood estimation. According to Hu and Bentler's (1999), the goodness-of-fit of the *a priori* models was estimated with the following fit indices: the overall χ^2^ statistics, Comparative Fit Index (CFI) > 0.90, Tucker Lewis Index (TLI) > 0.90, Standardized Root Mean Squared Residual (SRMR) ≤ 0.08, Root Mean Squared Error of Approximation (RMSEA) < 0.08.

A CFA was conducted for the total sample and separately for three age groups to test the model fit of the BRIEF2 three-factor and nine-factor models. The relevant information on these eight models is presented in Table 5.

**Table 5:** Summary of the 3-factor and 9-factor models fit indexes for the Russian version of the BRIEF2 teacher forms.

**Model**	**χ^2^**	**Df**	**CFI**	**TLI**	**SRMR**	**RMSEA 90% CI)**
***BRIEF2 3 factors***						
5-year-old children	40.2^**^	24	0.973	0.959	0.027	0.113 (0.04–0.17)
6-year-old children	44.1^**^	24	0.966	0.949	0.051	0.110 (0.05–0.16)
7-year-old children	106^***^	24	0.859	0.788	0.081	0.259 (0.21–0.31)
Total sample	100^***^	24	0.956	0.935	0.027	0.136 (0.10–0.16)
***BRIEF2 9 factors***						
5-year-old children	8743^***^	1674	0.201	0.155	0.146	0.282 (0.26–0.28)
6-year-old children	5518^***^	1674	0.327	0.288	0.164	0.182 (0.17–0.18)
7-year-old children	13310^***^	1674	0.108	0.056	0.369	0.369 (0.36–0.37)
Total sample	4677^***^	1674	0.633	0.612	0.108	0.102 (0.09–0.10)

** *p* < 0.01.

*** *p* < 0.001.

The results of the CFA indicate the best model fit for the three-factor model compared to the nine-factor models (Table 4). Furthermore, the underlying factor structure of the Russian version of the BRIEF2 Teacher Forms varied across different age groups. Thus, the three-factor model showed a good fit, especially for the samples of children aged 5 and 6 years, suggesting that the three-index model was applicable to the Russian version of the BRIEF2 Teacher Forms for both ages. All fit indices in these models responded to the thresholds except RMSEA (the RMSEA >0.08 indicates the insufficient extent to which the model matches the true model with its 90% confidence interval). The three-factor model for the samples of children aged 7 years and the total sample is not accurate enough in terms of goodness-of-fit. However, this factor solution is a better representation of the data than other models.

## Discussion

The objective of this study is to analyze the psychometric properties of the BRIEF2 Russian version in a sample of typically developing children in preschool and primary school settings. Reliability (internal consistency and interrater reliability) and validity (the underlying factor structure) of the BRIEF2 Russian version were analyzed as the main psychometric properties.

The internal consistency of the subscales of the BRIEF2 Russian questionnaire was heterogeneous among the three age groups. The highest consistency of the scales is observed when completing the BRIEF2 for children aged 5 years. In this age group, the internal consistency of all subscales was good or acceptable. When the form was completed for children aged 6 years, internal consistency was good or acceptable for all subscales except Initiative (0.57). Finally, in the sample of children aged 7 years (the first grade of school in Russia), the internal consistencies were, in general, lower than that in the previous two age groups but still good or acceptable for all subscales except Initiative (0.41). To summarize, the internal consistency of all subscales was adequate for reliability except for the Initiate. Internal consistency of the Initiate subscales was unacceptable among children aged 6 and 7 years and questionably acceptable in the sample of children aged 5 years. Furthermore, the internal consistency results on the three composite indices (BRI, ERI, and CRI) and the Global Executive Composite (GEC) have indicated adequate reliability (from 0.87 to 0.95).

The interrater reliability analysis results showed that the level of agreement between teachers when evaluating a child decreases slightly as children age. This is observed in the lower levels of agreement when assessing children aged 6 years compared to children aged 5 years. Yet, the two teachers' ratings of most subscales (except the Plan subscale for 5-year-olds and the Initiate subscale for 6-year-olds) were sufficiently consistent.

The confirmatory factor analysis (CFA) results of a construct validity study indicate the best model fit for the three-factor model compared to the nine-factor models. The underlying factor structure obtained in the present study suggests that the Russian version of the BRIEF2 Teacher Forms reflects three types of difficulties rather than nine. This result coincides with Jacobson et al. (2020), who found three factors in a large sample of children aged 5 to 18 years. Thus, the results indicate that clinical interpretation of the Russian version of BRIEF2 is more appropriate at the level of indices (BRI, ERI, and CRI) rather than subscales.

The sample of this study covered children aged 5 to 7 years to assess the applicability of the Russian-language version of BRIEF2 over existing differences in the age period of the transition from ECEC to primary school in the Russian educational context. It was shown through reliability and CFA analyses that the dimensional structure of the Russian version of BRIEF2 was different for children aged 5, 6, and 7 years. Both internal and interrater reliability and CFA indices of the goodness-of-fit of the a priori model decreased from younger to older children. While the 3-factor model showed a very good fit for the samples at ages 5 and 6 years, the fit indices were poorer at age 7. At the age of 7 in Russia, children enter first grade (see Introduction). This may indicate that the school setting in the Russian education system differs from the preschool setting to such an extent that the children's behavior can no longer be validly assessed using BRIEF2.

### Conclusion

The results of the present study confirm that the Russian version of the BRIEF2 Teacher Form can be used as a valid tool to assess EF in children aged 5 and 6 years based on observations of their behavior in kindergarten settings. However, three indices (BRI, ERI, and CRI) should be used instead of nine BRIEF2 subscales for clinical interpretation. For children aged 7 years, the BRIEF2 was a rather insufficiently valid tool for assessing EF based on CFA results.

### Limitations

From a future research perspective, we plan to analyze the external validity of the BRIEF2 using a battery of NEPSY-II tests to individually assess children's main EF skills (Korkman et al., 2007). The other important direction for further research should address the limitations of this study. The limitations of the present study are mainly related to sampling. A larger sample may help to obtain more reliable results, and psychometric evaluation of the Russian-language version of the BRIEF2 in a clinical sample is needed to determine its sensitivity to EF assessment in normative and clinical samples. The other limitation of the study is the fact that two teachers assessed children aged 5 and 6 years, while children aged 7 years were assessed by only one teacher. This difference is caused by the peculiarities of the educational system in the country of the study.

## Data availability statement

The raw data supporting the conclusions of this article will be made available by the authors, without undue reservation.

## Ethics statement

The studies involving humans were approved by the Ethics Committee of Faculty of Psychology at Lomonosov Moscow State University. The studies were conducted in accordance with the local legislation and institutional requirements. Written informed consent for participation in this study was provided by the participants' legal guardians/next of kin.

## Author contributions

MG: Formal analysis, Software, Validation, Writing—original draft. MA: Data curation, Formal analysis, Investigation, Writing—review and editing. KT: Data curation, Investigation, Software, Writing—review and editing. YZ: Conceptualization, Funding acquisition, Project administration, Resources, Supervision, Writing—review and editing.
